# Graphene Oxide Sheets Decorated with Octahedral Molybdenum Cluster Complexes for Enhanced Photoinactivation of *Staphylococcus aureus*

**DOI:** 10.1021/acs.inorgchem.3c01502

**Published:** 2023-08-23

**Authors:** Régis Guégan, Xiaoxue Cheng, Xiang Huang, Zuzana Němečková, Michaela Kubáňová, Jaroslav Zelenka, Tomáš Ruml, Fabien Grasset, Yoshiyuki Sugahara, Kamil Lang, Kaplan Kirakci

**Affiliations:** † Global Center for Science and Engineering, Waseda University, 3-4-1 Okubo, Shinjuku-ku, Tokyo 169-8555, Japan; ‡ Interfaces, Confinement, Matériaux et Nanostructures ICMN-UMR 7374, CNRS-Université d’Orléans, 1 Rue de la Férollerie, Orléans 45100, France; § Department of Applied Chemistry, Faculty of Science and Engineering, Waseda University, 3-4-1 Okubo, Shinjuku-ku, Tokyo 169-8555, Japan; ∥ Institute of Inorganic Chemistry of the Czech Academy of Sciences, Husinec-Řež 250 68, Czech Republic; ⊥ Department of Biochemistry and Microbiology, University of Chemistry and Technology Prague, Praha 166 28, Czech Republic; # Univ Rennes, CNRS, Institut des Sciences Chimiques de Rennes (ISCR)-UMR 6226, Rennes 35000, France; ∇ CNRS-Saint-Gobain-NIMS, IRL3629, Laboratory for Innovative Key Materials and Structures (LINK), National Institute for Materials Science, 1-1 Namiki, Tsukuba 305-0044, Japan; ○ Kagami Memorial Institute for Materials Science and Technology, Waseda University, 2-8-26 Nishiwaseda, Shinjuku-ku, Tokyo 169-0051, Japan

## Abstract

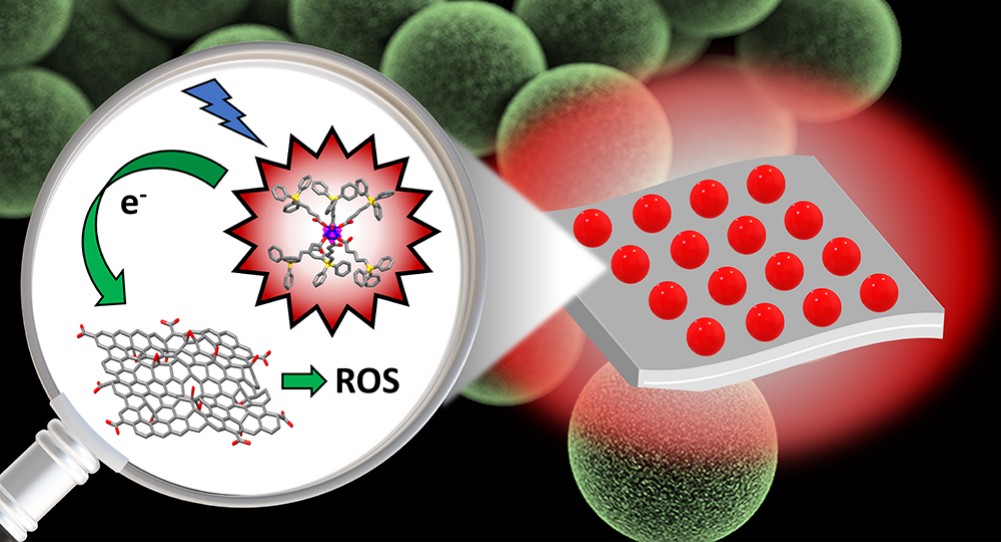

The emergence of multidrug-resistant microbial pathogens poses a significant threat, severely limiting the options for effective antibiotic therapy. This challenge can be overcome through the photoinactivation of pathogenic bacteria using materials generating reactive oxygen species upon exposure to visible light. These species target vital components of living cells, significantly reducing the likelihood of resistance development by the targeted pathogens. In our research, we have developed a nanocomposite material consisting of an aqueous colloidal suspension of graphene oxide sheets adorned with nanoaggregates of octahedral molybdenum cluster complexes. The negative charge of the graphene oxide and the positive charge of the nanoaggregates promoted their electrostatic interaction in aqueous medium and close cohesion between the colloids. Upon illumination with blue light, the colloidal system exerted a potent antibacterial effect against planktonic cultures of *Staphylococcus aureus* largely surpassing the individual contributions of the components. The underlying mechanism behind this phenomenon lies in the photoinduced electron transfer from the nanoaggregates of the cluster complexes to the graphene oxide sheets, which triggers the generation of reactive oxygen species. Thus, leveraging the unique properties of graphene oxide and light-harvesting octahedral molybdenum cluster complexes can open more effective and resilient antibacterial strategies.

## Abstract 2

We have developed a nanocomposite material consisting of an aqueous colloidal suspension of graphene oxide sheets adorned with nanoaggregates of octahedral molybdenum cluster complexes. The underlying mechanism behind this phenomenon lies in the photoinduced electron transfer from the nanoaggregates of the cluster complexes to the graphene oxide sheets, which triggers the generation of reactive oxygen species. Thus, leveraging the unique properties of graphene oxide and light-harvesting octahedral molybdenum cluster complexes can open more effective and resilient antibacterial strategies.

## Introduction

Microbial pathogens exhibiting multidrug-resistance constitute a serious hazard, especially to cancer and immunocompromised patients, and restrict the choices for adequate antibiotic therapy.^1,2^ Among the many possible ways to inactivate pathogens, their photoinactivation by materials able to produce reactive oxygen species (ROS) upon visible-light irradiation represents an elegant way to achieve this goal, as ROS attack several critical components of living cells, thus limiting the emergence of resistance to the treatment.

In this respect, octahedral molybdenum cluster complexes are efficient singlet oxygen photosensitizers and have recently demonstrated major potential as molecular antibacterial agents or for the design of light-triggered disinfecting surfaces.^3−9^ These clusters are stabilized by eight inner ligands, usually iodine atoms and six labile apical ligands of inorganic or organic nature. Upon excitation from the UV up to the green light, the resulting complexes exhibit long-lived triplet states that relax via a red-NIR phosphorescence or by energy transfer to molecular oxygen to form the highly reactive singlet oxygen O_2_(^1^Δ_g_).^10−12^ Several studies have reported the effect of these complexes for the photoinactivation of planktonic cultures and biofilms of both Gram-negative and Gram-positive bacteria.^3−9^ When compared to organic photosensitisers, these cluster complexes are less prone to self-quenching of their luminescent properties due to aggregation at high concentrations or photobleaching which can limit the efficiency and robustness of their derived materials.^7^

Graphene oxide (GO) sheets, prepared from the etching and oxidation of graphite, exhibit outstanding properties: large specific surface area and electrical and mechanical properties that tackle the interests of both the scientific and industrial communities.^13−17^ While the inclusion of oxygen atoms within carbon sheets modulates their electrical properties, it also confers a hydrophilic behavior to GO, making them dispersible in aqueous media as a supporting phase, for e.g., complexes or nanoparticles to prepare nanocomposite materials.^18−22^ In addition to the hydrophilic moieties, GO displays unaltered hydrophobic graphene-like patches which represent many reaction/adsorption sites for the association with other colloids. As in the case of other carbon-based nanomaterials (carbon nanotubes, etc.), GO sheets exhibit antibacterial properties, the origin of which comes from the physical presence of the sheets as well as their chemical activities.^13,16,17^ Indeed, it has been recognized that GO sheets wrap around bacteria causing irreversible physical damage to the cell membranes while chemically increasing the cellular oxidative stress, leading to the end of the bacterial development.^13^

The association of photosensitizing organic compounds such as hypocrellin A to GO sheets leads to nanocomposites showing long-time effects for the eradication of cancer cells.^23^ In this case, GO sheets act as a preservation matrix for hypocrellin A that could generate O_2_(^1^Δ_g_) on a long-time scale. In another study, the association of porphyrins with GO imparts the nanocomposites with antibacterial properties.^24^ The mechanism behind the antibacterial activity was assigned to fast electron transfer from the excited singlet states of porphyrin to the conduction band of GO, resulting in the production of ROS by the sheets.^24^ Thus, we surmise that the association of such cluster complexes with GO could lead to enhancement of photoinactivation of bacteria. In fact, various composites of these cluster complexes with GO have already been utilized with promising results for applications such as photocatalytic water reduction, photodegradation of organics, or gas sensing, but so far, no such composite material has been studied for its antibacterial properties.^25−29^

**Figure 1:**
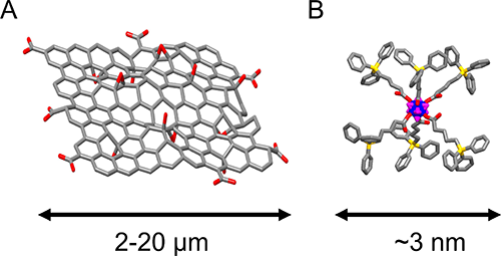
Schematic representation of (A) GO sheets and (B) positively charged Mo_6_ complex [Mo_6_I_8_(OCOC_4_H_8_PPh_3_)_6_]^4+^. Color codes: carbon (gray), oxygen (red), phosphorus (yellow), molybdenum (blue), iodine (magenta).

This research work describes the association of the cluster compound, [Mo_6_I_8_(OCOC_4_H_8_PPh_3_)_6_]Br_4_ (Mo_6_), with GO sheets (Figure 1) for the preparation of nanocomposites with antibacterial properties. Dispersed in dimethyl sulfoxide to provide a homogeneous dispersion of the components and long-term durability for Mo_6_, and then added to aqueous medium, GO sheets act as a template or supporting phase of Mo_6_ in the form of nanoaggregates interacting mainly via electrostatic forces. After a careful characterization of the morphology, cohesion, and structure of the nanocomposites as well as of their photophysical properties by various complementary techniques, their bactericide properties against *Staphylococcus aureus* are demonstrated through a comparative study of the efficiency of the whole individual materials.

## Results and Discussion

### Preparation and Characterization of GO

**Figure 2:**
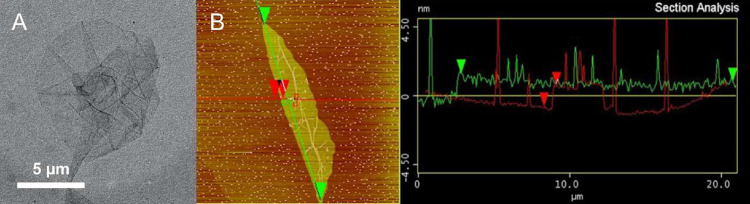
Characterization of the GO sheets by TEM (A) and AFM with the corresponding section analysis (B).

GO sheets were prepared using the modified Hummer’s method based on the oxidation and exfoliation of graphite.^30^ As demonstrated by several studies, the diversity of the chemical landscape of GO sheets and their 2D-surfactant character (Pickering emulsion) allow for dispersion in many polar and nonpolar organic solvents.^14,31^ However, the use of dimethyl sulfoxide (DMSO), a polar solvent often used in biological experiments, failed so far to give stable GO dispersions.^31^ In the aforementioned study, the sheets were dried in a vacuum oven which favored the re-stacking of the sheets into the initial graphite-like layered structure, slowing down their subsequent exfoliation and leading to the formation of GO agglomerates and sedimentation of the graphite oxide. In the present study, the prepared GO sheets, displaying a similar oxidation rate, were lyophilized which allowed for an easy dispersion in DMSO and water at a concentration of 1 mg mL^–1^, higher than that previously tested using a wide range of solvents (0.5 mg mL^–1^) (Figure S1 in the Supporting Information).^31^ This feature was confirmed by transmission electron microscopy (TEM) and atomic force microscopy (AFM) observations, indicating the absence of re-stacking and aggregation of the sheets as the images showed a majority of individual sheets with a lateral size in the 2–20 μm range and a thickness close to 1 nm (Figures 2 and S2 in the Supporting Information), an important point to consider for future applications based on single carbon nanomaterials.

**Figure 3:**
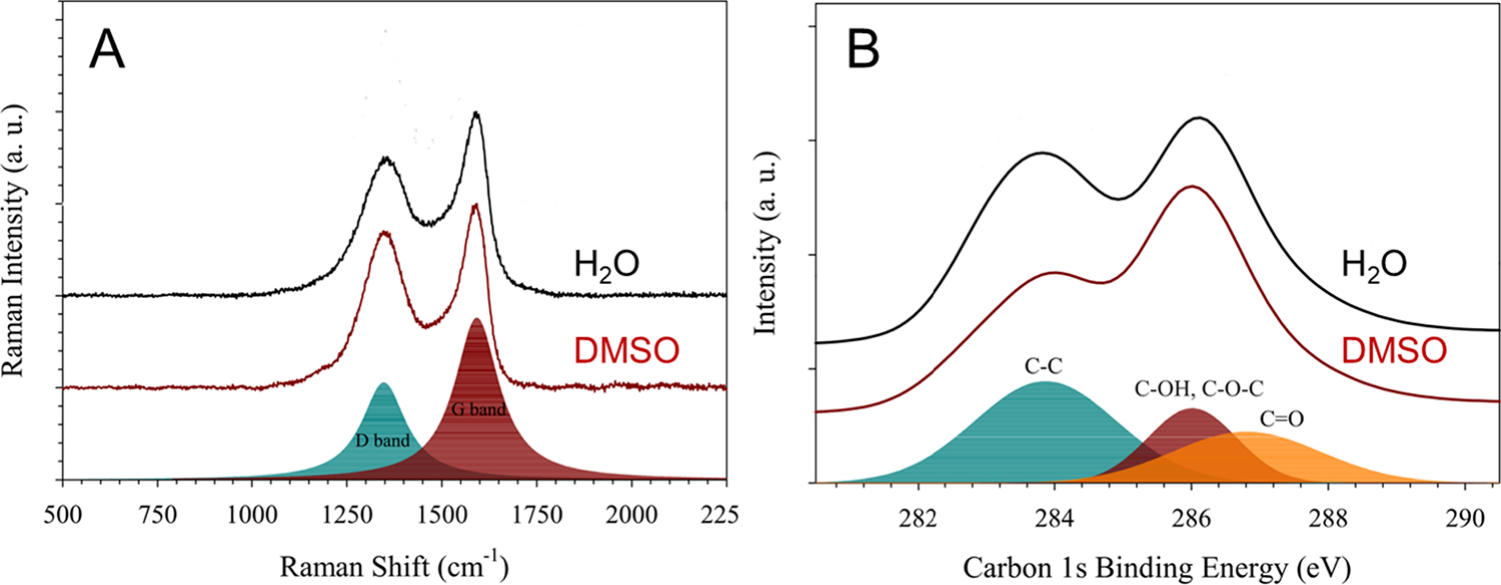
Raman scattering (A) and XPS (B) of the GO sheets after being dispersed in water (black) and in DMSO (red).

The GO sheets, after being dispersed in water or DMSO, were collected and characterized by Raman scattering and XPS (Figure 3) to assess the possible chemical changes after their immersion in DMSO. As expected, the Raman spectra of the GO showed two characteristic bands of graphitized materials, i.e., the D band reflecting structural defects due to inclusion of oxygen atoms in the carbon structure or holes and the G band representative of the original graphite organization.^32^ The intensity ratio *I*_D_/*I*_G_ is an indicator of the degree of graphitization of the GO sheets and remained the same for the GO sheets (0.87) before and after immersion in water or DMSO (Table 1). It underlines the absence of any modification and physical structural alteration of the sheets after the freeze-drying operation. However, the XPS spectrum of the DMSO immersed GO seemed to display a higher proportion of C=O double bonds at the expense of a lowering of C–C bonds (graphite areas), which is contradictory with the Raman data that suggested the same proportion of graphitic domains. Since DMSO is a rather viscous solvent with a high boiling point, it is likely that some DMSO molecules remain on the surface of the GO sheets and the C–S band could overlap with the C 1s spectrum of the GO as well as the resulting distribution of the various bonds. Nevertheless, the characterization by Raman scattering clearly evidences that the GO sheets were not altered neither physically nor chemically after their lyophilization and dispersion in DMSO.

**Table 1:** Component Area Percentages of the GO Sheets Dispersed in H_2_O and DMSO and Corresponding Ratios of the G Band over the D Band

sample	C–C	C–OC, C–OH	C=O	*I*_D_/*I*_G_ (Raman)
GO (H_2_O)	50.2%	42.6%	7.2%	0.87
GO (DMSO)	44.6%	32.8%	22.6%	0.87

### Preparation and Characterization of GO/Mo_6_ Nanocomposites

The dispersibility of the GO sheets in DMSO after a freeze-drying operation, with the formation of stable dispersions, opens new routes for the preparation of nanocomposites. Leaning against this strategy, the nanocomposites were prepared by mixing the lyophilized GO and [Mo_6_I_8_(OCOC_4_H_8_PPh_3_)_6_]Br_4_ in DMSO for 24 h under magnetic stirring. The Mo_6_ complex suffers from a lack of stability and poor solubility in aqueous media where its apical ligands are replaced within days, but it is well soluble in DMSO where the hydrolytic process is greatly slowed down.^3^ Thus, DMSO solutions of the Mo_6_ complex did not exhibit any significant aggregation by showing an average size of 3.1 ± 1 nm (*Z*-average = 6.0 nm, PDI = 0.20) (Figure S3 in the Supporting Information), which is consistent with the size of the isolated Mo_6_ complex. Due to the targeted antibacterial application, the GO/Mo_6_ nanocomposite was studied in the form of an aqueous colloidal dispersion which was obtained by adding the aliquots of the DMSO dispersion (1 mg mL^–1^) to deionized water (DMSO/H_2_O, 1% v/v).

**Figure 4:**
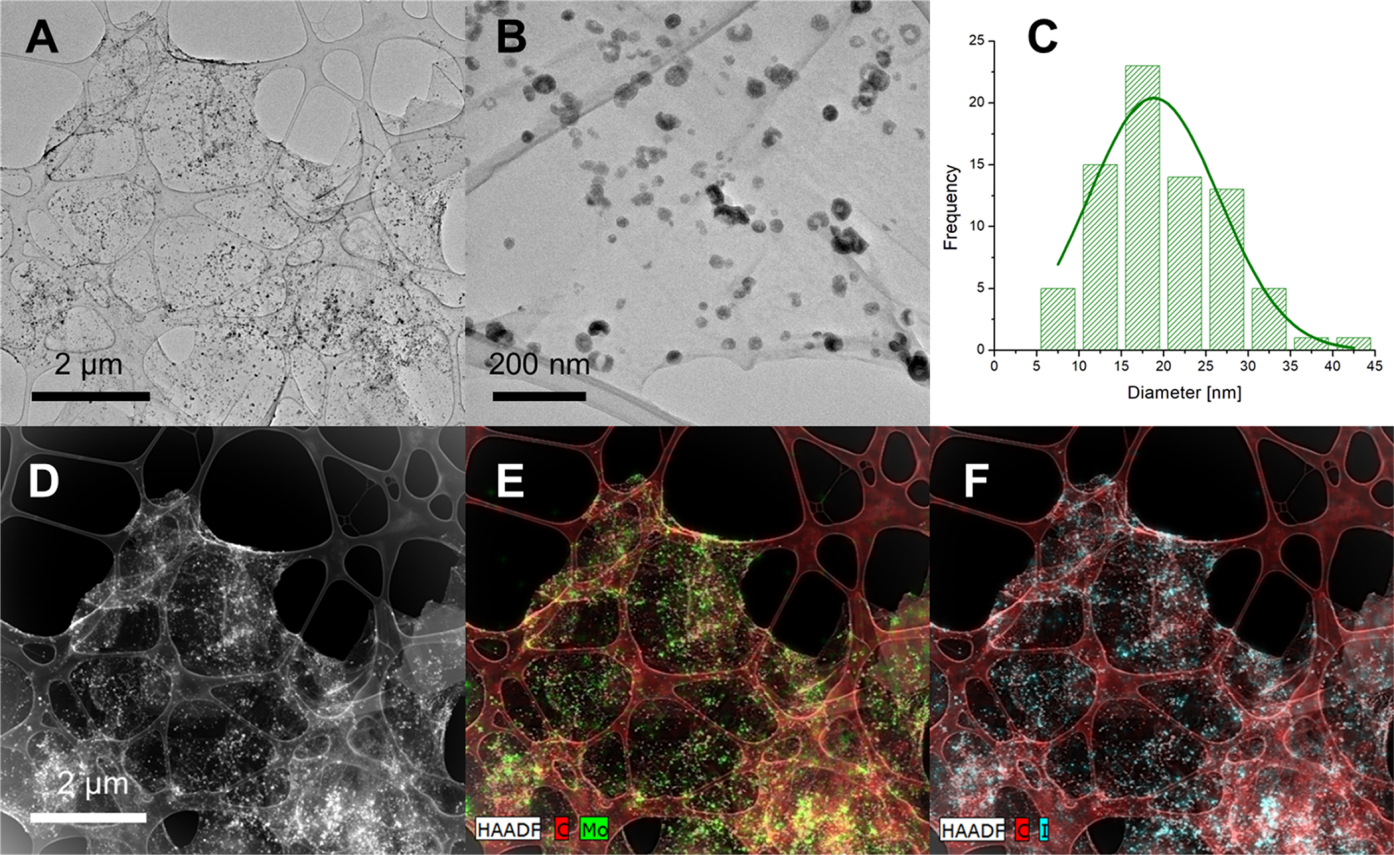
TEM images of GO/Mo_6_ in the bright field (A, B) with the corresponding particle size distribution of Mo_6_ nanoaggregates (C) and in the dark field (D) with the C, Mo (E) and C, I (F) HAADF elemental mapping.

TEM of a drop of the resulting aqueous dispersion of GO/Mo_6_ deposited on a TEM grid documented GO sheets decorated by nanoaggregates with an average size of 19 ± 8 nm and made of Mo_6_ as demonstrated by the HAADF elemental mapping of Mo and I, (Figures 4 and S4 in the Supporting Information). The Mo_6_ nanoaggregates immobilized on the surface of GO are apparently formed during the addition of the DMSO dispersion to H_2_O. Indeed, dynamic light scattering of pure Mo_6_ in water evidenced nanoaggregates with an average size by the number of 31 ± 10 nm (*Z*-average = 110 nm, PDI = 0.37) (Figure S3 in the Supporting Information), reminiscent of the nanoparticles of other pure cluster compounds obtained via the solvent displacement method.^33−35^ X-ray powder diffraction patterns of GO/Mo_6_ featured a very broad peak at approximately 7°, demonstrating the amorphous character of the nanocomposite material (Figure S5 in the Supporting Information). ICP-MS analysis of dried GO/Mo_6_, isolated by centrifugation of the water dispersion, revealed a molybdenum content of 68.1 mg g^–1^ slightly lower than the theoretical content of 70.5 mg g^–1^, indicating that the majority of the nanoaggregates of Mo_6_ are immobilized at the surface of GO. This feature is in agreement with the absorption spectra of the supernatant after centrifugation of the GO/Mo_6_ water dispersion, which evidenced only a minute amount of free Mo_6_ (Figure S6 in the Supporting Information). The zeta potentials of the aqueous dispersions of GO and Mo_6_ amounted to −34 ± 13 and 8 ± 6 mV, respectively, in accordance with the ionization of the hydroxyl and carboxylic groups of GO and the presence of positive charges endowed by the apical ligands of Mo_6_ at the surface of the nanoaggregates (Figure S7 in the Supporting Information). The aqueous dispersion of GO/Mo_6_ displayed a negative zeta potential of −19 ± 8 mV and the zeta potential distribution was characterized by a single peak located in the negative range of the zeta potential values. In accordance with the abovementioned characterizations, these results confirm that, in aqueous medium, the majority of the positively charged Mo_6_ nanoaggregates is immobilized at the GO surface, where they partially compensate for its negative charge.

Despite the mild conditions used for the preparation of the nanocomposite in DMSO and the bulkiness of the apical ligands making them poorly labile, it is still possible that an apical ligand exchange could occur in this solvent, resulting in bonding of cluster complexes to the surface of GO. In this case, we should expect a homogeneous coverage of the GO surface by Mo_6_ considering the high degree of structural defects due to inclusion of oxygen atoms evidenced by Raman spectroscopy. However, TEM images of the nanocomposite clearly show the noncontinuous distribution of nanoaggregates of Mo_6_ at the surface of GO (Figures 4 and S4). Thus, the mechanism of the formation of the GO/Mo_6_ nanocomposite can be tentatively ascribed to the precipitation of positively charged nanoaggregates of hydrophobic Mo_6_ after mixing the DMSO dispersions of GO and Mo_6_ in water, followed by the immobilization of the nanoaggregates at the surface of GO mainly via electrostatic interactions. Indeed, it is unlikely that a significant ligand exchange with hydroxyl or carboxylates function of the GO could occur during the short period (minutes to hours) between the transfer of the DMSO dispersion to water and the measurement of the nanocomposite characteristics.

**Figure 5:**
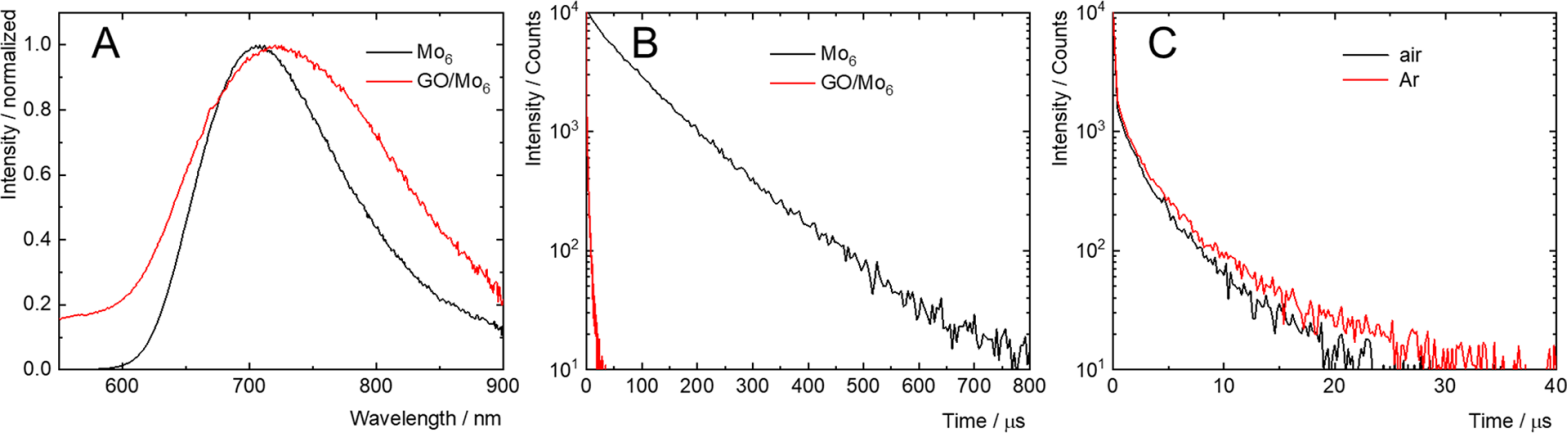
(A) Normalized luminescence spectra of Mo_6_ (black) and GO/Mo_6_ (red) in argon-saturated water, excitation was at 400 nm. (B) Luminescence decay kinetics of Mo_6_ (black) and GO/Mo_6_ (red) in argon-saturated water. Excitation was at 405 nm and emission at 700 nm. (C) Luminescence decay kinetics of GO/Mo_6_ in air- (black) and argon-(red) saturated water dispersions. Excitation was at 405 nm and emission at 700 nm.

The luminescence of Mo_6_ complexes is influenced by their ligands as well as their immediate environment and can be taken as a sensitive indicator of the extent of interaction between Mo_6_ and GO. Therefore, the photophysical properties of water dispersions of the nanocomposites and their individual components, freshly prepared in the same way as for DLS experiments, were investigated and are summarized in Table 2. The absorption spectrum of GO/Mo_6_ was essentially a composite of its individual components GO and Mo_6_ showing typical absorption bands of Mo_6_ complexes in the UV/blue region and the featureless UV–vis absorption of GO extending to the red region (Figure S8 in the Supporting Information). When compared to the free Mo_6_ complex, the luminescence band of GO/Mo_6_ was broader and the maximum was red-shifted from 706 nm for Mo_6_ to approximately 722 nm for GO/Mo_6_ (Figure 5A). A drastic decrease in the luminescence intensity was also noticed, as also evidenced by a significant drop of luminescence quantum yields from 0.24 for Mo_6_ to 0.01 for GO/Mo_6_. Similarly, the average luminescence lifetime in argon-saturated dispersions decreased from 88 μs for Mo_6_ to 2.4 μs for GO/Mo_6_ (Figure 5B). It is worth noting that as-prepared DMSO dispersions of Mo_6_ and GO/Mo_6_ displayed comparable luminescence spectra, indicating that the cluster complex was chemically unchanged prior to addition to aqueous medium as hydrolysis would be characterized by a red-shift of the emission maximum (Figure S9 in the Supporting Information). In addition, the kinetics of hydrolysis of Mo_6_ in aqueous medium were already reported showing that the wide majority of the cluster complexes remained intact after 24 h.^3^ Thus, hydrolysis of Mo_6_ complex in DMSO or immediately after addition to aqueous medium was excluded to account for the strong decrease in emission quantum yield and lifetime of GO/Mo_6_ in water relative to pure Mo_6_. Instead, the presented results point out at electronic interactions between the immobilized nanoaggregates of Mo_6_ and GO, leading to the quenching of their excited states.

**Table 2:** Photophysical Parameters of Water Dispersions of GO, Mo_6_, and GO/Mo_6_ at Room Temperature^a^

sample	λ_L_/nm	τ_T_/μs	τ_air_/μs	Φ_L_
GO	670	0.7 ns^b^	0.7 ns^b^	<0.01
Mo_6_	706	88^b^	29^b^	0.24
GO/Mo_6_	722	2.4^b^	2.3^b^	0.01

a λ_L_ is the maximum of luminescence emission bands (excited at 400 nm); τ_T_ and τ_air_ are the average luminescence lifetimes in argon- (oxygen-free) and air-saturated water, respectively (recorded at 700 nm, excited at 405 nm); Φ_L_ is the luminescence quantum yield in argon-saturated solutions (excitation at 400 nm, experimental error of Φ_L_ is ±0.01).

b The amplitude average lifetimes obtained by the biexponential analysis of corresponding kinetic curves.

Contrasting with free Mo_6_ nanoaggregates (Figure S10 in the Supporting Information), there was negligible quenching of the triplet states by oxygen after their deposition onto the GO sheets as evidenced by the average luminescence lifetimes of GO/Mo_6_ in air- and argon-saturated water (2.4 and 2.3 μs, respectively, Table 2) (Figure 5C). This result also indicates fast competitive quenching of the triplet states of deposited Mo_6_, probably associated with photoinduced electron transfer between Mo_6_ and the GO sheets and suggest a poor production of O_2_(^1^Δ_g_) by deposited Mo_6_ upon irradiation. Note that a very weak red emission from a GO water dispersion was observed when exciting at 400 nm. It was characterized by a broad band with a maximum at approximately 670 nm and an emission lifetime of 0.7 ns, which is consistent with the reports on GO (Table 2 and Figure S11 in the Supporting Information).^16^

**Figure 6:**
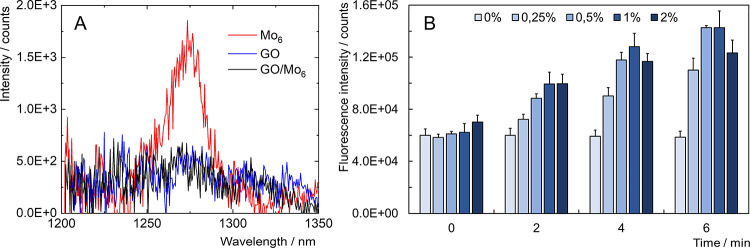
(A) Singlet oxygen phosphorescence band of Mo_6_ (red), GO (blue), and GO/Mo_6_ (black) in oxygen-saturated water. (B) Evaluation of the oxidation of the DCF-DA probe by various concentrations of GO/Mo_6_ in water (0, 0.25, 0.5, 1, 2%) under various blue-light irradiation time (460 nm). Bars labeled 0% are control experiments in the absence of GO/Mo_6_.

The inhibition of O_2_(^1^Δ_g_) production by deposited nanoaggregates of Mo_6_ in GO/Mo_6_ was confirmed by measuring the weak phosphorescent signal of O_2_(^1^Δ_g_) centered at approximately 1270 nm. Indeed, while Mo_6_ showed a typical phosphorescence band of O_2_(^1^Δ_g_), as previously reported for this complex,^3^ both GO and GO/Mo_6_ did not display any detectable signal in the same spectral region, confirming that GO and GO/Mo_6_ did not produce an appreciable amount of O_2_(^1^Δ_g_) under these conditions, as previously reported for GO (Figure 6A).^36^ In order to evaluate the capacity of the nanocomposite to generate other reactive oxygen species, such as superoxide, hydrogen peroxide, or hydroxyl radical, we employed an oxidation probe, namely, 2′,7′-dichlorofluorescein diacetate (DCF-DA). It is a chemically reduced form of fluorescein commonly used as an indicator for ROS. DCF-DA was added to water dispersions containing various concentrations of GO/Mo_6_ (0, 0.25, 0.5, 1, and 2 w/w %) followed by irradiation with 460 nm light. The probe was oxidized thus becoming fluorescent, and the amount of oxidation was dose- and time-dependent, evidencing the capacity of GO/Mo_6_ to photogenerate reactive oxygen species (Figure 6B).

Overall, the observed changes in the zeta potentials and luminescence parameters when comparing Mo_6_ and GO/Mo_6_ point out at strong electrostatic interactions between the GO sheets and the nanoaggregates of Mo_6_ in aqueous medium. The quenching of the luminescence of Mo_6_ by GO inhibits the formation of O_2_(^1^Δ_g_). This feature appears to be related to a photoinduced electron transfer from the excited states of the Mo_6_ complex to the conduction band of GO and leads to the formation of ROS, probably superoxide, hydrogen peroxide, or hydroxyl radical, as previously reported in the literature.^37−39^

### Bactericide Properties of the GO/Mo_6_ Nanocomposites

The photoinactivation properties of GO/Mo_6_ and its individual components were evaluated against planktonic culture of *S. aureus*, a Gram-positive spherically shaped bacterium. Despite acting as a commensal of the human microbiota, it can be the cause of skin and respiratory infections as well as food poisoning. *S. aureus* constitutes one of the leading lethal pathogens and is associated with antimicrobial resistance, making it an ideal target for photodynamic inactivation.^1,2^ The cultures of *S. aureus* were incubated as planktonic cells with GO, Mo_6_, and GO/Mo_6_ dispersions and illuminated with 460 nm light.

**Figure 7:**
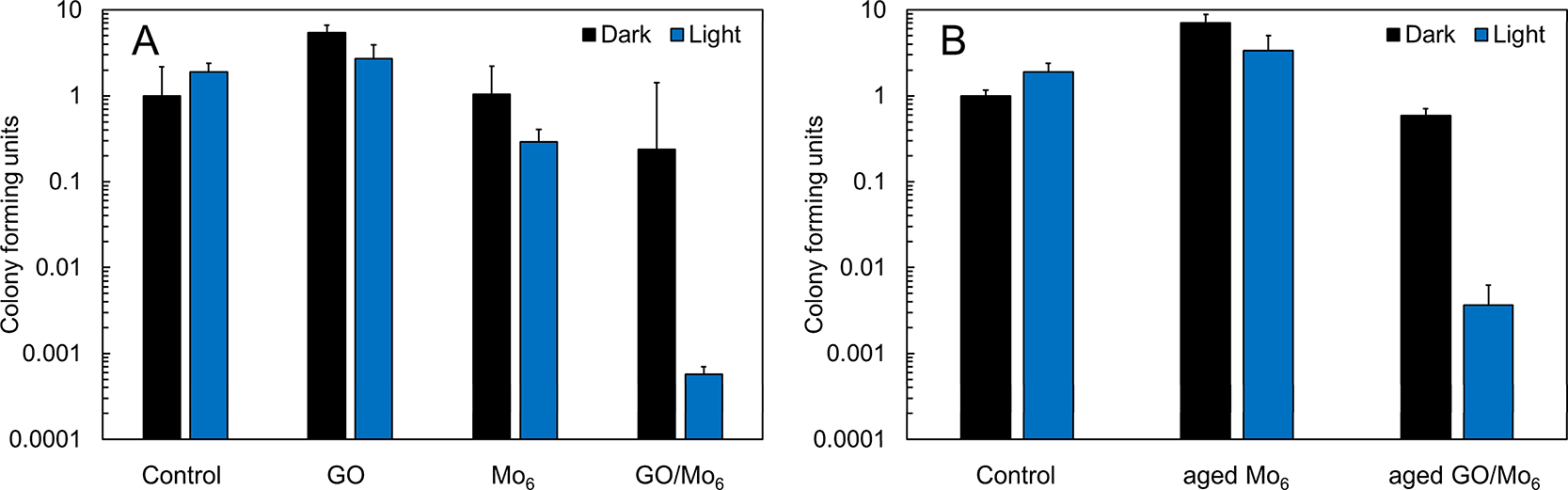
Photoinactivation of *Staphylococcus aureus* planktonic cultures upon 460 nm irradiation in the presence of (A) GO, Mo_6_, or GO/Mo_6_ (0.01 g L^–1^) and (B) aged Mo_6_ and GO/Mo_6_. Dark controls are represented with black bars and bars labeled control are experiments in the absence of GO, Mo_6_, or GO/Mo_6_.

In the absence of light, GO, Mo_6_, and GO/Mo_6_ were nontoxic against *S. aureus* at the used concentration of 0.01 mg mL^–1^ (Figure 7A). GO sheets were previously reported to exert an antibacterial effect on *S. aureus*; however, in our case, we did not observe such effect possibly due to lower concentration and bigger lateral size for the GO used in our experiments.^40^ Upon irradiation at 460 nm, GO remained nontoxic, while Mo_6_ displayed a mild photoinactivation with a relative decrease of the CFU to 29 ± 12% compared to the control, an effect comparable to previous investigations on the antibacterial activity of this complex.^3^ Under the same conditions, the photoinactivation ability of GO/Mo_6_ was remarkable with a relative decrease of the CFU to 0.06 ± 0.01% compared to the irradiated control, i.e., the killing was almost three orders of magnitude more efficient than for Mo_6_ only (Figure 7A). Photoinduced intracellular oxidative stress was evaluated by incubating *S. aureus* with 0.01 mg/mL of GO/Mo_6_ or its individual components in the presence of DCF-DA as an oxidation probe (Figure S12 in the Supporting Information). While GO/Mo_6_ clearly showed increased photoinduced ROS production, when compared to pure GO, the oxidative ability of Mo_6_ alone was higher than that of GO/Mo_6_. Possibly, this feature which is in contradiction with the photoinactivation experiments, arises from different subcellular localization of the materials. Indeed, DCF-DA and Mo_6_ should be internalized while GO/Mo_6_ should be located at the surface of the bacteria as previously described for GO.^13^ Aging of the DMSO solutions of Mo_6_ for 6 months led to a total loss of the photoinactivation ability due to hydrolysis of the complex evidently triggered by water traces in DMSO, as previously reported for this complex.^3^ On the other hand, aged GO/Mo_6_ nanocomposites maintained a high photoinactivation efficiency with a CFU of 0.37 ± 0.26% (Figure 7B). The robustness of the system that allows for long-term storage in DMSO suggests a protection of the GO sheets against the hydrolysis of the clusters. Overall, the observed enhancement of the efficiency of the photoinactivation of *S. aureus* was clearly related to a synergistic effect of both components, GO and Mo_6_, probably originating from photoinduced electron transfer from the triplet states of excited Mo_6_ to GO, leading to the formation of reactive oxygen species.

## Experimental Section

### Reagents and General Procedures

Compound [{Mo_6_I_8_}(OCOC_4_H_8_PPh_3_)_6_]Br_4_ (Mo_6_) was prepared according to a previously published procedure.^3^ Molybdenum, iodine, sodium methanolate, and (4-carboxybutyl)triphenylphosphonium bromide were obtained from Sigma Aldrich and used as received. Solvents for synthesis were purchased from Penta (Czech Republic) and dried over molecular sieves (3 Å). The following reagents were used for the synthesis of graphene oxide without purification: graphite powder (Wako, 98.0%), sulfuric acid (Wako, 95%), sodium nitrate (Wako, 98.0%), potassium permanganate (Kanto, 99.3%), hydrogen peroxide (Kanto, 30.0%), hydrochloric acid (Wako, 35.0–37.0%).

Raman measurements were recorded with an in Via Reflex spectrometer operating at 732 nm. XPS measurements were performed with a JEOL JPS-9010TR spectrometer. Atomic force microscopy (AFM) images were obtained using the AFM multimode function on a Digital Instruments AFM. Images of the nanoparticles were acquired by a FEI Talos F200X transmission electron microscope (Thermo Fisher Scientific). Size distributions and corresponding zeta potentials were determined by dynamic light scattering (DLS) on a particle size analyzer Zetasizer Nano ZS (Malvern, UK). Powder X-ray diffraction (XRD) patterns were recorded using a PANalytical X’Pert PRO diffractometer in the transmission setup equipped with a conventional Cu X-ray tube (40 kV, 30 mA). The molybdenum content of GO/Mo_6_ was measured by inductively coupled plasma mass spectrometry (ICP-MS, PerkinElmer, Concord, ON, Canada). The sample was isolated by centrifugation of the GO/Mo_6_ water dispersion (10,000 rpm, 5 min) and drying of the solid under reduced pressure for 24 h. Quantification was carried out via external calibration. The ICP-MS measurement conditions were as follows: RF power 1.1 kW, nebulizer gas flow rate 0.76 L min^–1^, auxiliary gas flow rate 1 L min^–1^, plasma gas flow rate 11 L min^–1^, measured isotope ^98^Mo as an analyte, and ^115^In as an internal standard. UV–vis absorption spectra and luminescence properties were measured on an FLS1000 spectrometer (Edinburgh Instruments, UK) using a cooled PMT-980 photon detection module (Edinburgh Instruments, UK). Aqueous dispersions (0.1 mg mL^–1^ GO/Mo_6_) were saturated with air or argon to assure different oxygen concentrations for phosphorescence analyses. The FLS1000 spectrometer was also used for time-resolved phosphorescence measurements (λ_exc_ = 405 nm, VPLED Series) and the recorded decay curves were fitted to exponential functions by the Fluoracle software (v. 2.13.2, Edinburgh Instruments, UK). Phosphorescence quantum yields of the samples were recorded using a Quantaurus QY C11347-1 spectrometer (Hamamatsu, Japan). Singlet oxygen phosphorescence was measured on a Fluorolog 3 spectrometer using a Hamamatsu H10330-45 photomultiplier. In this case, aqueous dispersions of similar absorbance were saturated with oxygen to magnify phosphorescence signals of O_2_(^1^Δ_g_). Evaluation of the photoinduced oxidation of 2′,7′-dichlorofluorescein diacetate (DCF-DA) was performed by adding 10 μM of DCF-DA to the GO/Mo_6_ water dispersion (0.01 mg mL^–1^), illuminating with a 12 × 10 W LED source (Cameo) (460 nm, 18 mW cm^–2^) for 0, 2, 4, and 6 min and measuring the fluorescence of the whole suspension at 488/525 nm.

### Preparation of Graphene Oxide

Graphene oxide (GO) was synthesized based on Hummer’s method. Sulfuric acid (92 mL), sodium nitrate (2 g), and graphite (2 g) were added to a 500 mL beaker and stirred for 30 min in an ice bath. After adding potassium permanganate (10 g), the mixture was stirred at 35 °C for 40 min. After adding pure water (92 mL) again in the ice bath, the mixture was stirred at 70 °C for 20 min. Then, pure water (200 mL) was added, and 30% hydrogen peroxide was added little by little until the absence of formation of bubbles. The precipitate obtained by centrifugation (3000 rpm, 10 min) was washed with 5% hydrochloric acid and pure water. Centrifugation (15,000 rpm, 40 min) was performed to remove ions such as sulfate ions that cause a decrease in pH, and the supernatant was discarded. The obtained precipitate was dispersed in pure water and centrifuged to collect the supernatant (8000 rpm, 30 min). Finally, the GO dispersion was concentrated by centrifugation (15,000 rpm, 40 min) to reach a concentration of 1 g L^–1^.

### Preparation of GO/Mo_6_ Dispersions

The colloidal dispersions of GO were freeze-dried to collect dehydrated GO. The freeze-drying method prevents any restacking of the sheets and facilitates their dispersion in other solvents. Then, colloidal dispersions of GO in DMSO were prepared at a concentration of 1 g L^–1^. [{Mo_6_I_8_}(OCOC_4_H_8_PPh_3_)_6_]Br_4_, dissolved in DMSO solvent at a concentration of 1 g L^–1^, was added to the GO dispersions. The resulting dispersions were stirred at 200 rpm for 1 day and directly used for the formation of water dispersions (DMSO–H_2_O v:v = 0.01).

### Photoinactivation of Bacteria and ROS Determination

Bacterial samples of *Staphylococcus aureus* were cultivated at 37 °C and stored at 4 °C either in the liquid Luria–Bertani (LB) medium or on LB agar. The stock solutions of Mo_6_ were prepared in DMSO. All experiments were performed in triplicates. The stock inoculum of *Staphylococcus aureus* was prepared by diluting bacteria in water and standardizing suspension to 1 McF. A 100 μL aliquot of the inoculum was taken and mixed with GO, Mo_6_, or GO/Mo_6_ to a final concentration of 0.01 mg mL^–1^. The samples were incubated for 2 h in the dark at laboratory temperature and afterward irradiated with a 12 × 10 W LED source (Cameo) (460 nm, 18 mW cm^–2^, 15 min). For quantification of inactivated bacteria, the Miles and Misra method on LB agar was used. Evaluation of the photoinduced oxidation of DCF-DA was performed by treating inoculum of *S. aureus* at 1 McF in water with 0.01 mg mL^–1^ of GO, Mo_6_, or GO/Mo_6_ (1% DMSO) for 2 h, adding DCF-DA in DMSO to a final concentration 10 μM, illuminating with a 12 × 10 W LED source (Cameo) (460 nm, 18 mW cm^–2^) for 0, 5, and 10 min and measuring the fluorescence of the whole suspension at 488/525 nm. The control experiments were performed using the same concentration of DMSO (1% v/v in final suspension) introduced by the addition of GO, Mo_6,_ or GO/Mo_6_ in the dark or upon irradiation. The effect of aging of dispersions on the antibacterial activity was performed using 6 months-old DMSO dispersions of Mo_6_ and GO/Mo_6_.

## Conclusions

We have designed a nanocomposite material based on graphene oxide sheets and a Mo_6_ compound. Lyophilization of the GO sheets after preparation allowed for the formation of stable colloidal dispersions of GO. Upon the addition of the DMSO colloidal dispersions of GO and Mo_6_ to water, the formation of positively charged nanoaggregates of Mo_6_ occurred, followed by their immobilization on the surface of the GO sheets via electrostatic interaction as demonstrated by DLS, TEM, ICP-MS, and absorption and luminescence spectroscopy. In agreement with the electronic properties of GO when combined with photoactive sensitizers, photoinduced electron transfer from excited states of Mo_6_ to GO was suggested for explaining the dramatic quenching of the triplet states when Mo_6_ is associated with GO. This phenomenon also led to the inhibition of singlet oxygen formation by Mo_6_. Injection of electrons from Mo_6_ into the conduction band of GO led to the formation of reactive oxygen species, possibly superoxide, hydrogen peroxide, or hydroxyl radicals, providing a robust antibacterial activity against *S. aureus* far stronger than that of the isolated components of the nanocomposite. The observation of the synergistic effect paves the way for the design of nanomaterials with enhanced photooxidative properties which have potential applications in life and environmental sciences. The easy preparation of the described material as well as the use of inexpensive components suggest relevant potential for scaling up the production of this nanocomposite material. From a prospective point of view, we believe that the properties of such nanocomposite can be further tuned and optimized by the careful choice of apical ligands of cluster complex and by varying the size, oxidation rate, or chemically modifying the surface of the GO sheets. Also, the incorporation of the nanocomposite into functional materials such as surfaces and membranes could expand the potential for antibacterial and environmental applications.

## Supporting Information Available

Photographs of GO dispersions; TEM and AFM of GO; DLS of Mo_6_; zeta potentials of GO, Mo_6_, and GO/Mo_6_; powder XRD of GO/Mo_6_; absorption spectra; luminescence spectra; luminescence decay kinetics; and photoinduced ROS production of GO, Mo_6_, and GO/Mo_6_ (PDF)
