# Supplementary material for: Graphene Oxide Sheets Decorated with Octahedral Molybdenum Cluster Complexes for Enhanced Photoinactivation of Staphylococcus aureus
Source: Inorg Chem. 2023 Aug 23;62(35):14243–51. doi: 10.1021/acs.inorgchem.3c01502 (PMC10481373; doi:10.1021/acs.inorgchem.3c01502)

Supporting Information

Graphene oxide sheets decorated with octahedral molybdenum cluster complexes for enhanced photoinactivation of *Staphylococcus aureus*

Régis Guégan[1,2*], Xiaoxue Cheng[3], Xiang Huang[3], Zuzana Němečková,[4] Michaela Kubanová[5], Jaroslav Zelenka[5], Tomas Ruml[5], Fabien Grasset[6], Yoshiyuki Sugahara[3,7], Kamil Lang[4], Kaplan Kirakci[4*]

[1]*Global Center for Science and Engineering, Waseda University, 3-4-1 Okubo, Shinjuku-ku, Tokyo 169-8555, Japan*

[2]*Interfaces, Confinement, Matériaux et Nanostructures ICMN-UMR 737, CNRS-Université d'Orléans, 1 Rue de la Férollerie, 45100 Orléans, France*

[3]*Department of Applied Chemistry, Waseda University, Faculty of Science and Engineering, 3-4-1 Okubo, Shinjuku-ku, Tokyo 169-8555, Japan*

[4]*Institute of Inorganic Chemistry of the Czech Academy of Sciences, 250 68 Husinec-Řež, Czech Republic*

[5]*Department of Biochemistry and Microbiology, University of Chemistry and Technology Prague, 166 28 Praha, Czech Republic*

[6]*Institut de Sciences Chimiques de Rennes, ISC-UMR 6226, CNRS-Université de Rennes 1, 263 avenue du Général Leclerc, 35700 Rennes, France*

[7]*Kagami Memorial Institute for Materials Science and Technology, Waseda University, 2-8-26 Nishiwaseda, Shinjuku-ku, Tokyo 169-0051, Japan*

**email: regis.guegan@univ-orleans.fr, kaplan@iic.cas.cz*

Content

Figure S1. Photographs of GO dispersions in H_2O and DMSO.

Figure S2. Characterization of the GO sheets by transmission electron microscopy and atomic force microscopy.

Figure S3. Size distributions of $[Mo_6I_8(OCOC_4H_8PPh_3)_6]Br_4$ (Mo_6) in DMSO and H_2O as obtained by DLS.

Figure S4. Transmission electron microscope images of GO/Mo_6.

Figure S5. Powder X-ray diffraction pattern of GO/Mo_6

Figure S6. Absorption spectra of GO/Mo_6 in water and the corresponding supernatant after centrifugation.

Figure S7. Zeta potential distributions of water dispersions of GO, Mo_6 and GO/Mo_6 as obtained by dynamic light scattering.

Figure S8. Absorption spectra of GO, Mo_6 and GO/Mo_6 water dispersions.

Figure S9. Luminescence spectra of Mo_6 and GO/Mo_6 in argon-saturated DMSO.

Figure S10. Luminescence decay kinetics of Mo_6 in air- and argon-saturated water.

Figure S11. Luminescence spectrum and decay kinetics of GO in water.

Figure S12. Photoinduced oxidative stress in *S. aureus* measured by DCF-DA method.

Figure S1. Visual appearance of the GO dispersions in H_2O (A) and DMSO (B) at a concentration of 1 mg mL^{-1}.

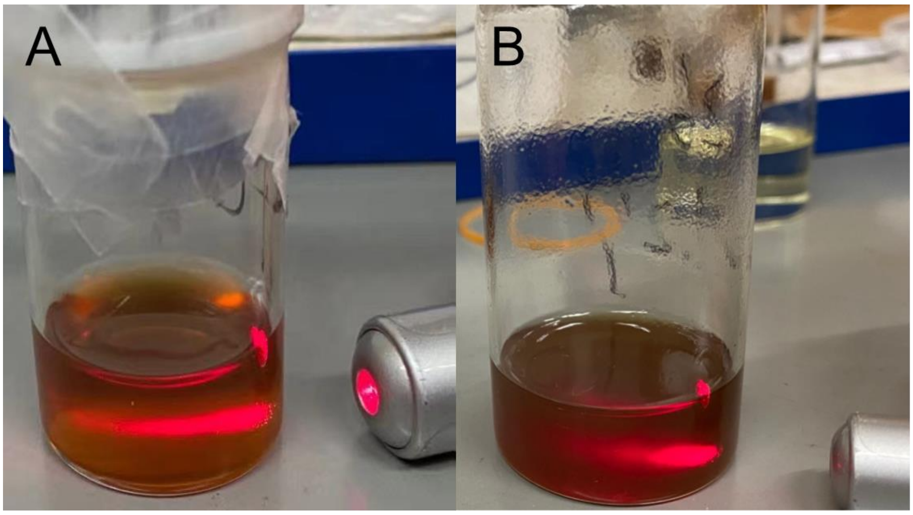

Figure S2. Characterization of the GO sheets by transmission electron microscopy (**A**) with a corresponding size distribution (**B**). Atomic force microscopy and section analysis of GO (**C**).

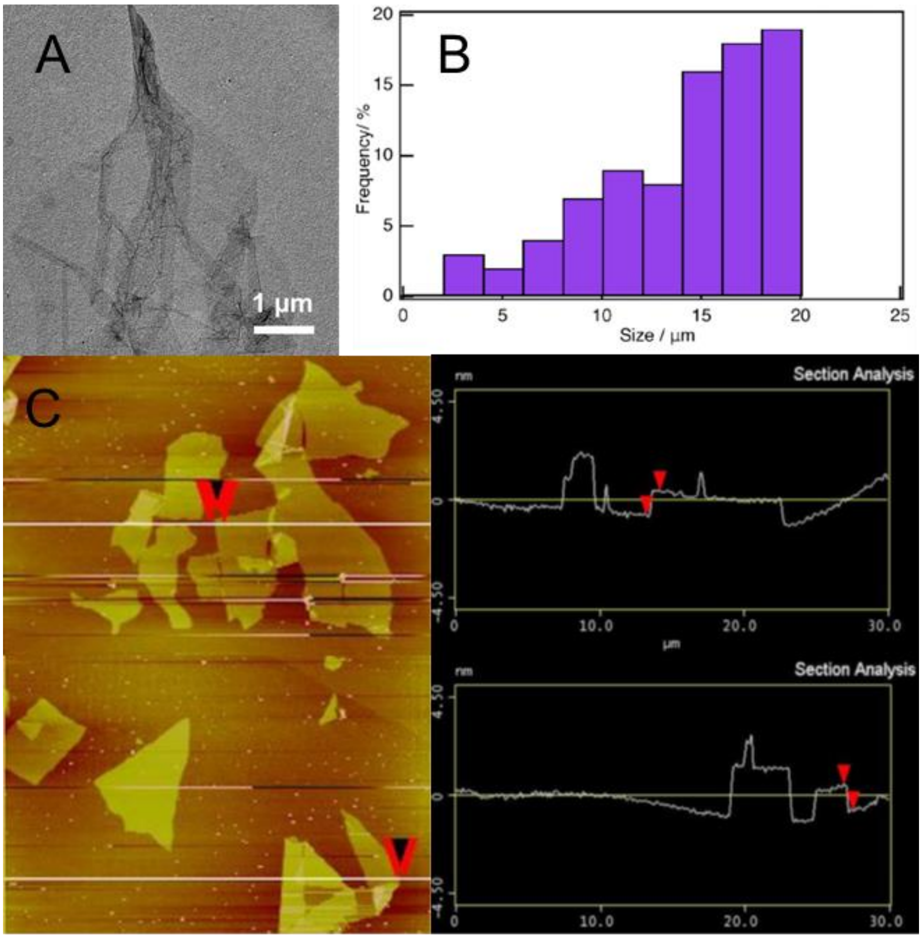

Figure S3. Size distributions by number (**A**) and by intensity (**B**) of $[Mo_6I_8(OCOC_4H_8PPh_3)_6]Br_4$ (Mo_6) in DMSO (green) and H_2O (red) as obtained by dynamic light scattering.

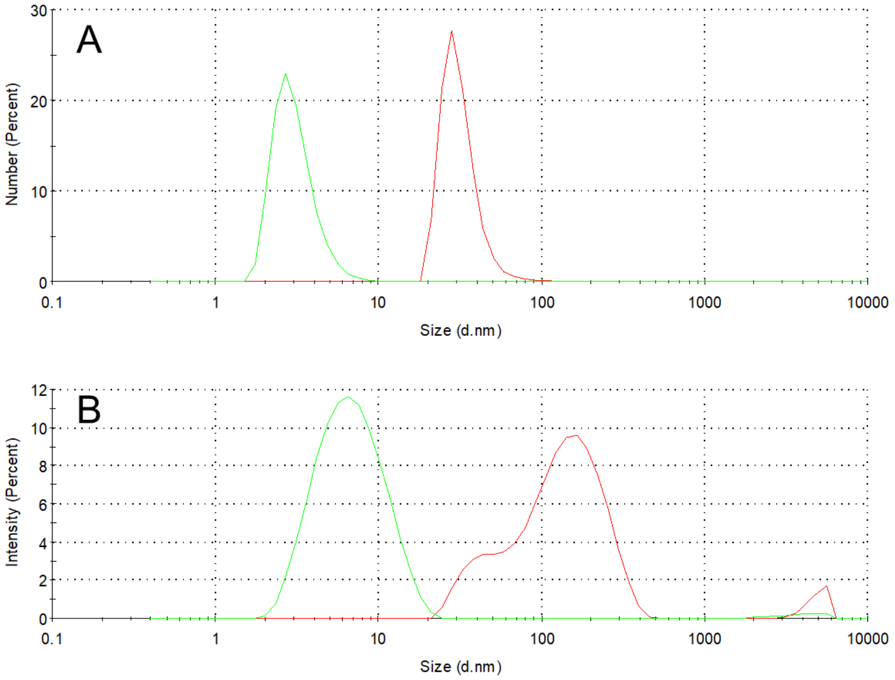

Figure S4. Transmission electron microscope images of GO/Mo_6 in the bright field (**A**) and in the dark field (**B**) with the C, Mo (**C**) and C, I (**D**) HAADF elemental mapping.

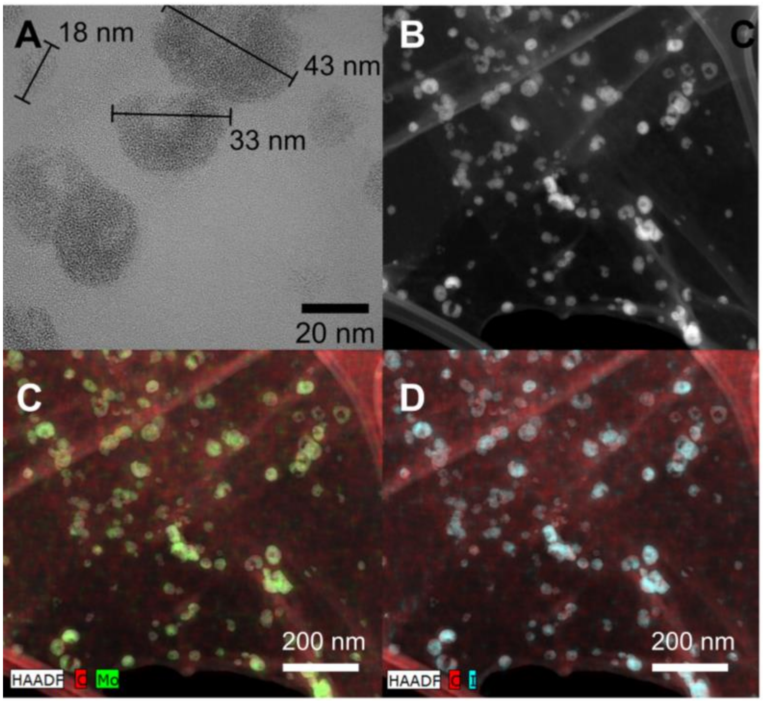

Figure S5. Powder X-ray diffraction pattern of GO/Mo_6 deposited on a Mylar® foil evidencing the amorphous character of the nanocomposite material.

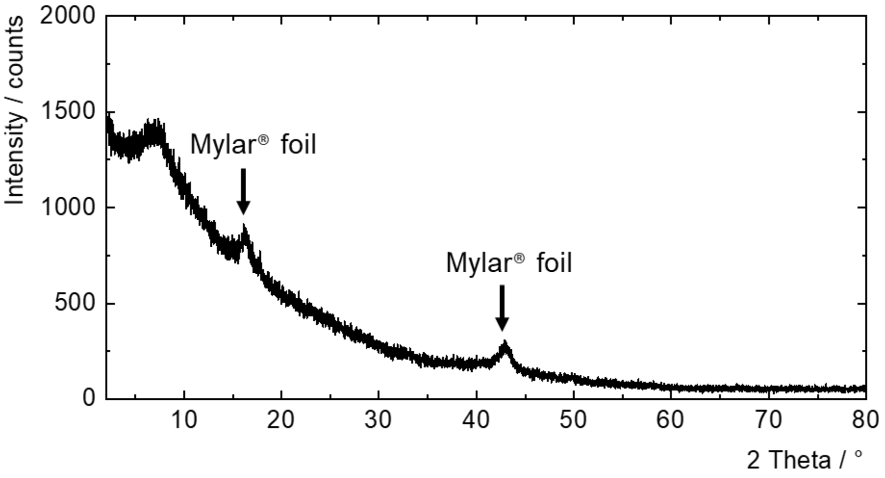

Figure S6. Absorption spectra of GO/Mo_6 in water (black) and the corresponding supernatant after centrifugation (10 000 rpm, 5 min) (red).

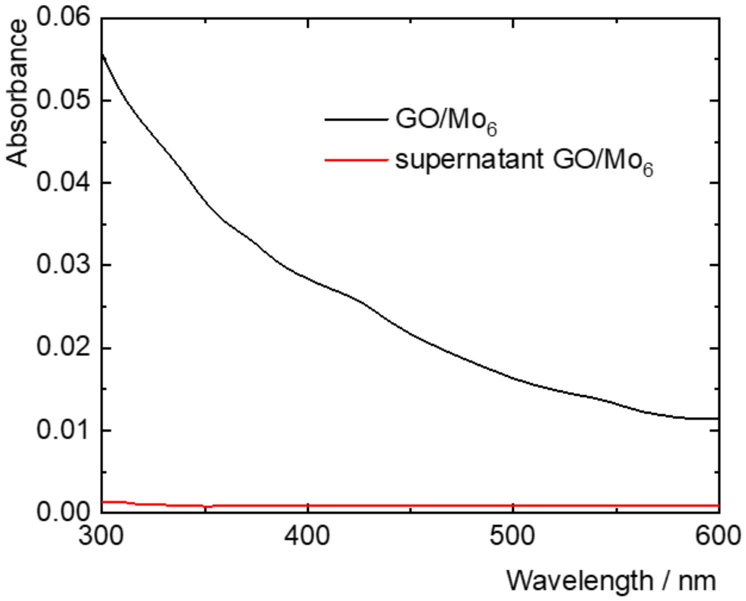

Figure S7. Zeta potential distributions of water dispersions of GO (blue), Mo_6 (black) and GO/Mo_6 (red) as obtained by dynamic light scattering.

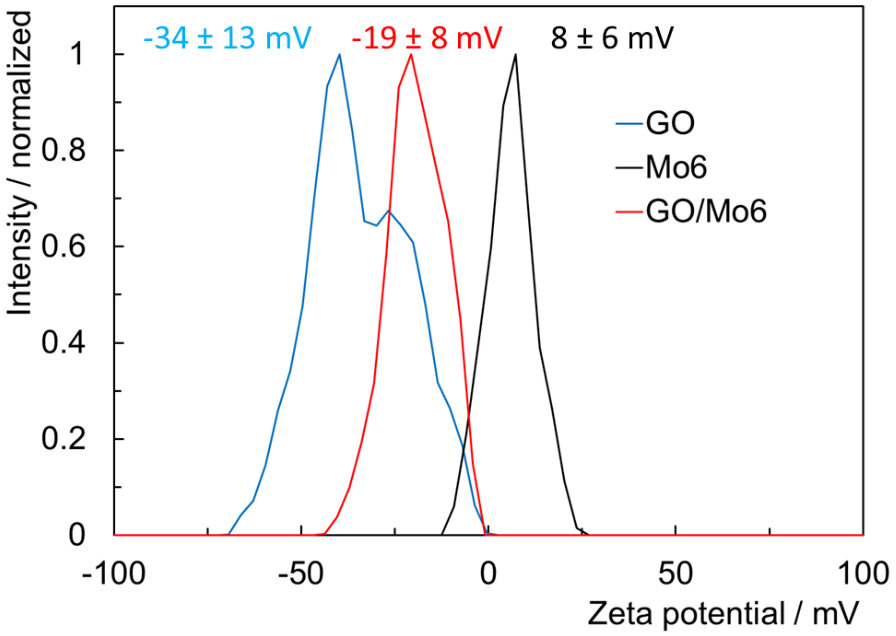

Figure S8. Absorption spectra of GO (blue), Mo_6 (black) and GO/Mo_6 (red) water dispersions.

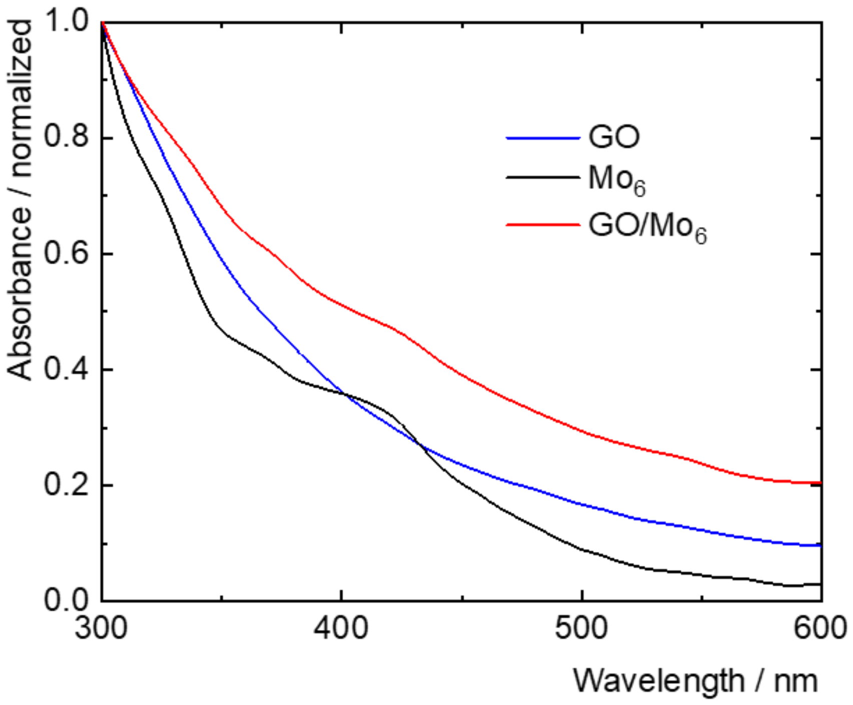

Figure S9. Luminescence spectra of Mo_6 and GO/Mo_6 in argon-saturated DMSO. Excitation was at 405 nm.

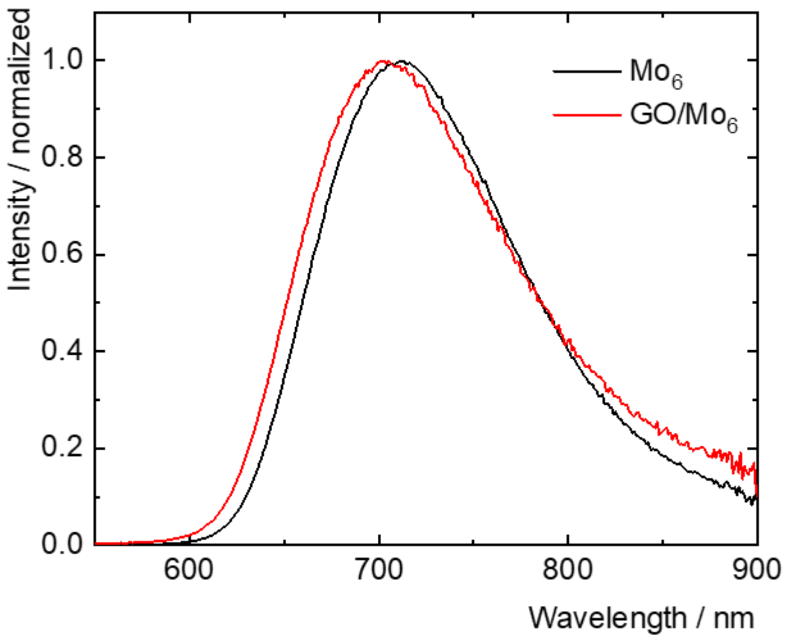

Figure S10. Luminescence decay kinetics of Mo_6 in air- and argon-saturated water. Excitation was at 405 nm and emission at 700 nm.

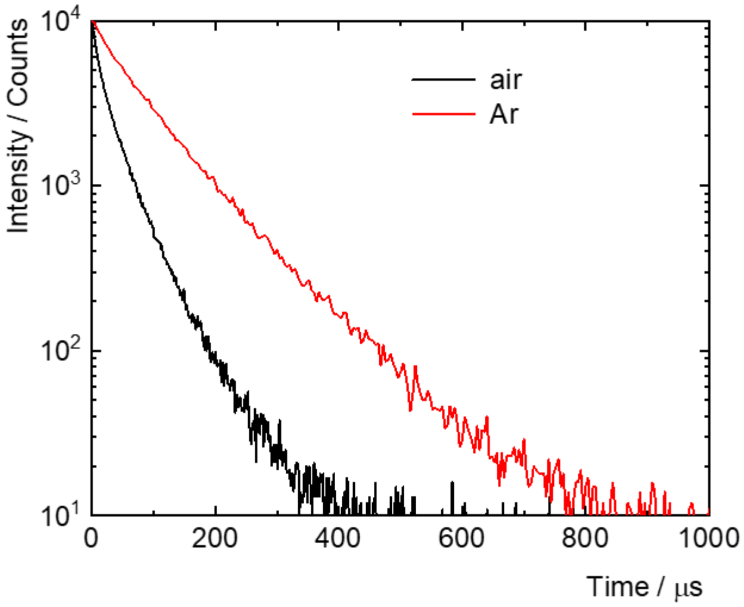

Figure S11. (**A**) Luminescence spectrum of GO in water upon excitation at 400 nm. (**B**) Luminescence decay kinetics of GO in water (red) with the corresponding excitation pulse profile. Excitation was at 405 nm and emission was collected at 700 nm.

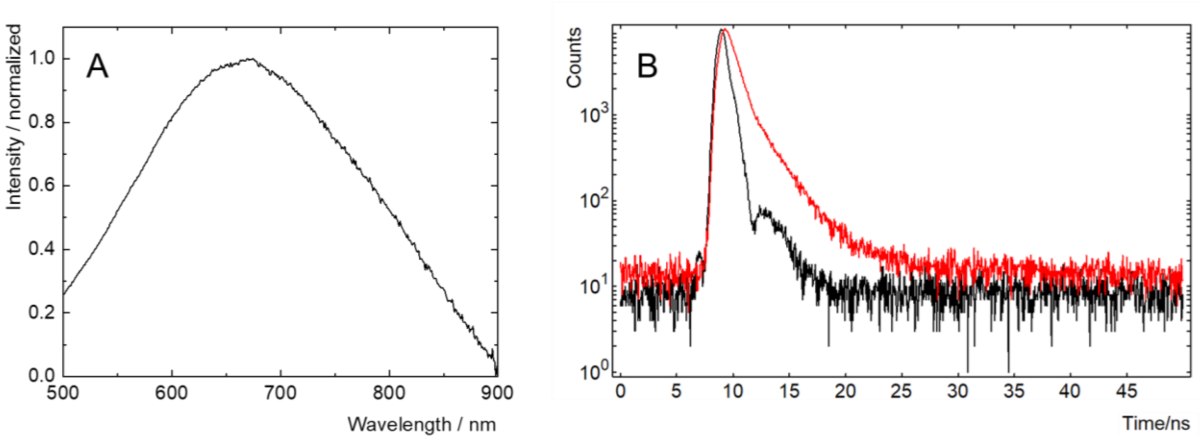

Figure S12 Evaluation of the oxidation of the DCF-DA probe under various blue-light irradiation time (460 nm) for *S. aureus* incubated with 0.01 mg mL^{-1} of GO/Mo_6, GO, or Mo_6. Bars labelled **Control** are control experiments in the absence of GO/Mo_6, GO, or Mo_6 during incubation.

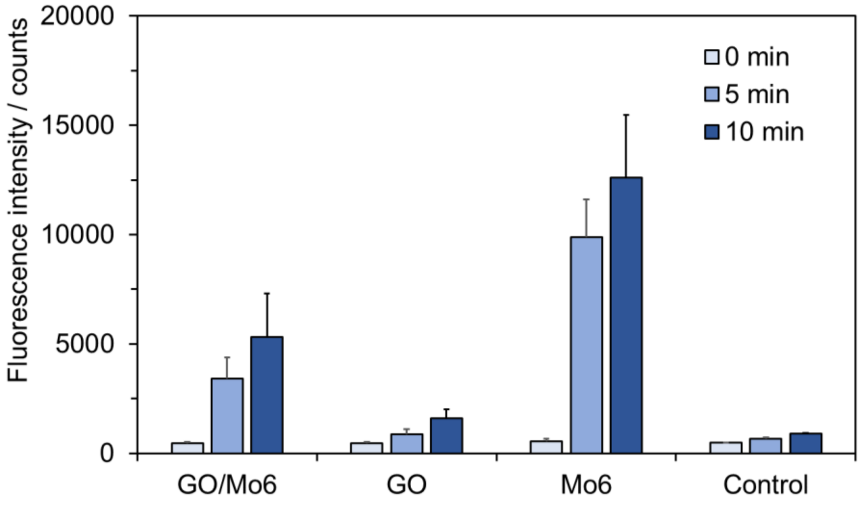

Supplement: Supplementary file 1 — ic3c01502_si_001.pdf [file ic3c01502_si_001.pdf]
